# Improved Resistive Switching Characteristics and Synaptic Functions of InZnO/SiO_2_ Bilayer Device

**DOI:** 10.3390/ma16237324

**Published:** 2023-11-24

**Authors:** Dongyeol Ju, Minsuk Koo, Sungjun Kim

**Affiliations:** 1 Division of Electronics and Electrical Engineering, Dongguk University, Seoul 04620, Republic of Korea; judongyeol0117@gmail.com; 2 Department of Computer Science and Engineering, Incheon National University, Incheon 22012, Republic of Korea

**Keywords:** neuromorphic computing, synaptic plasticity, spiking neural network, resistive switching, InZnO

## Abstract

This paper investigates the bipolar resistive switching and synaptic characteristics of IZO single-layer and IZO/SiO_2_ bilayer two-terminal memory devices. The chemical properties and structure of the device with a SiO_2_ layer are confirmed by x-ray photoemission spectroscopy (XPS) and transmission electron microscopy (TEM) imaging. The device with the SiO_2_ layer showed better memory characteristics with a low current level, as well as better cell-to-cell and cycle-to-cycle uniformity. Moreover, the neuromorphic applications of the IZO/SiO_2_ bilayer device are demonstrated by pulse response. Paired pulse facilitation, excitatory postsynaptic current, and pulse-width-dependent conductance changes are conducted by the coexistence of short- and long-term memory characteristics. Moreover, Hebbian rules are emulated to mimic biological synapse function. The result of potentiation, depression, spike-rate-dependent plasticity, and spike-time-dependent plasticity prove their favorable abilities for future applications in neuromorphic computing architecture.

## 1. Introduction

To overcome the scaling issue of present complementary metal-oxide-semiconductor (CMOS) technology and the bottleneck problem of Von Neumann computing architecture, various next-generation memory devices to implement neuromorphic computing architecture have emerged in recent years [1,2,3,4,5,6]. Because of its easy fabrication method, high switching speed, low operating voltage, and non-volatility, resistive random-access memory (RRAM) is regarded as the most promising choice for the next generation of memory [7,8,9,10,11,12,13,14,15,16,17,18,19]. Furthermore, several structures expandable to high density, such as 3D vertical structures and array structures, are easy to use due to their simple metal–insulator–metal structures [20,21,22,23,24,25,26,27].

The resistive switching phenomena of metal-oxide-based RRAM of the bipolar switching type are caused by the creation and rupture of a conducting filament in the insulating layer that connects the top and bottom electrodes [28,29,30,31,32,33,34,35,36]. When the conducting filament links the top and bottom electrodes under external voltage bias, a large current flows with decreasing resistance. Thus, the device switches from its initial resistance state to a low-resistance state (LRS), indicating the device is ‘on’. Alternatively, when an opposite polarity bias is employed, it causes a break in the conducting path, restricting the flow of the current and transitioning the device into a state of high resistance (HRS), effectively turning it ‘off’. Various metal oxides such as TaO_x_ [37], ZrO_2_ [38], ZnO [39,40], SiO_2_ [41,42], and HfO_x_ [43] have been widely studied for resistive switching. Uniformity poses a significant challenge in metal-oxide-based RRAM because of the sporadic creation and disruption of conducting filaments. One way to improve uniformity is by inserting a thin SiO_2_ layer, which prevents hard breakdown and enables repeated switching [44]. Among these, ZnO is gathering interest due to the suitable range of its bandgap (~3.37 eV at 300K) for resistive switching [45], good transparency [46], and abundant defects [47] for memory applications. Also, indium zinc oxide (IZO) is a popular semiconductor material applied in various devices such as thin film transistors (TFT) [48,49], oxide diodes [50], and sensors [51] due to its high thermal stability, low film stress, and high transparency [52,53,54]. However, most of the IZO applications in RRAM involve making transparent electrodes [55,56]. Although Hsu et al. discovered a bipolar resistive switching phenomenon in sol–gel IZO, its synaptic applications can be investigated further [57].

In this paper, we fabricated an IZO layer-based RRAM device using RF sputtering. Thin silicon oxide was introduced between the bottom electrode and the IZO layer to improve its resistive switching properties. In addition to an improvement in uniformity, the current level was reduced, improving its power consumption. Furthermore, the synaptic characteristics of the ITO/IZO/SiO_2_/TaN bilayer device were characterized by applying a pulse on the device. Due to the coexistence of short- and long-term memory characteristics, potentiation, depression, paired-pulse facilitation (PPF), and excitatory postsynaptic current (EPSC) were performed [58,59,60,61,62]. Finally, spike-rate-dependent plasticity (SRDP) and spike-time-dependent plasticity (STDP) of the Hebbian rule were emulated to investigate the ability of ITO/IZO/SiO_2_/TaN (tantalum nitride) stack to act as a synapse device [63,64,65].

## 2. Experimental Section

To fabricate the RRAM device, a SiO_2_/Si wafer was cleaned using acetone and isopropyl alcohol (IPA). Subsequently, a TaN bottom electrode with a thickness of 100 nm was sputtered onto the SiO_2_/Si wafer using DC sputtering. A Ta target of 99.99% purity was employed at a DC power of 65 W. The sputtering gas was a mixed gas of Ar (19 sccm) and N_2_ (1 sccm) with a deposition pressure of 5 mTorr. Next, for the IZO/SiO_2_ bilayer device, on top of the TaN layer, SiO_2_ was deposited using low-pressure chemical vapor deposition (LPCVD). The deposition occurred at 785 °C by reacting dichlorosilane (DCS, SiCl_2_H_2_, 40 sccm) and N_2_O (160 sccm). Then, for both devices, IZO 50 nm thick was deposited through a radio frequency (RF) sputter, with an RF power of 50 W. The deposition pressure was 2 mTorr, and the reactive gas consisted of Ar 10 sccm and O_2_ 1 sccm. Following the deposition of the IZO film, square patterns measuring 100 µm × 100 µm were created using photolithography. Subsequently, a indium tin oxide (ITO) top electrode 120 nm thick was deposited and shaped through a lift-off process using acetone after the RF sputter deposition of ITO, which employed a commercial ITO target of 99.99% purity, operated at an RF power of 80 W. The gas pressure stood at 3 mTorr with an Ar flow of 8 sccm. The electrical characteristics of both devices were assessed using the Keithley 4200-SCS semiconductor parameter analyzer and the 4225-PMU pulse measurement unit from Keithley Instruments in Cleveland, OH, USA. The bias was applied to the top electrode (ITO), while the bottom electrode (TaN) remained grounded. Additionally, the device’s schematic and its chemical properties were examined using X-ray photoelectron spectroscopy (XPS) and transmission electron microscopy (TEM) (Oxford Instruments, Tubney Woods, Abingdon, UK).

## 3. Results and Discussion

**Figure 1:**
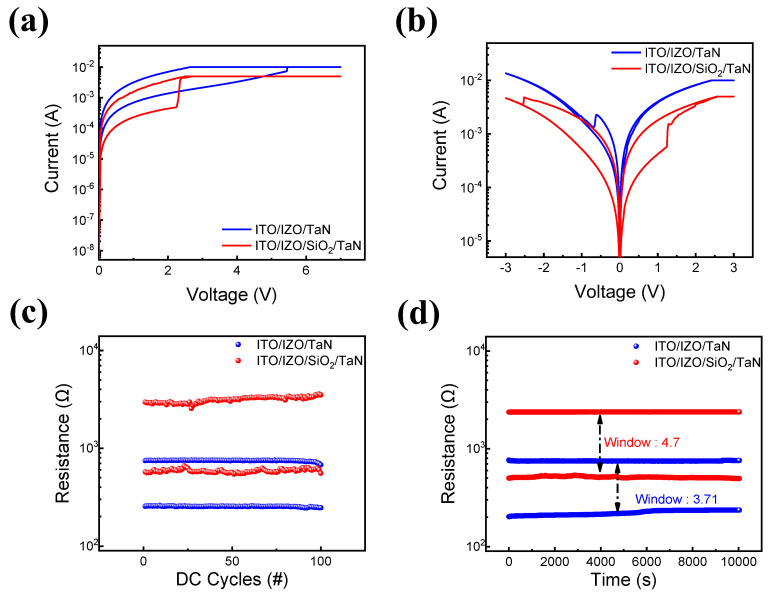
Resistive switching characteristics of devices with and without an SiO_2_ layer. (**a**) I–V curves of forming process; (**b**) I–V curves of set and reset processes. (**c**) Endurance characteristics; (**d**) retention test in HRS and LRS at a read voltage of −0.2 V.

Figure 1 shows the resistive switching characteristics of IZO-based RRAM devices. Figure 1a shows that both ITO/IZO/TaN single-layer and ITO/IZO/SiO_2_/TaN bilayer devices require a forming process to transition from an initial resistance state to an LRS [63,66]. The forming process is often known as the initial set or breakdown process, accumulating the defects in thin films under a higher voltage applied prior to the resistive switching phenomenon. The forming process is controlled by compliance currents of 10 mA and 5 mA for single-layer and bilayer devices, respectively. The I-V curves of the devices are shown in Figure 1b. Both devices were controlled at less than 3 V and −3 V for the set and reset processes. However, different compliance currents were needed in order to avoid a hard breakdown. For the single-layer IZO device, a high compliance current of 30 mA was needed. However, for the bilayer ITO/IZO/SiO_2_/TaN device, a relatively lower compliance current of 5 mA is required. The cycle-to-cycle endurance and retention characteristics of both devices were then examined. In Figure 1c,d, it is shown that both devices remained in their LRS and HRS states during 100 endurance cycles and 10^4^ s of retention time, with voltages of 3 and −3 V applied to trigger the resistive switching phenomenon and a read bias of −0.3 V. In both experiments, the bilayer ITO/IZO/SiO_2_/TaN device had a lower operating current level and a larger window compared to the ITO/IZO/TaN device. This phenomenon can be noted as the effect of inserting a thin SiO_2_ film, decreasing the on and off currents, and improving the device’s power consumption.

**Figure 2:**
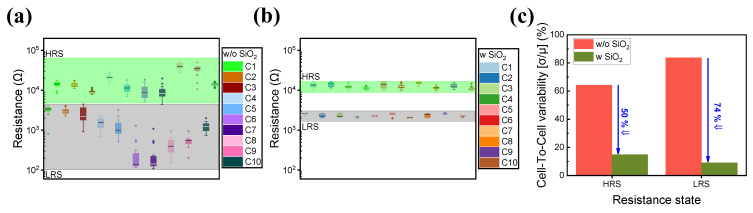
Cell-to-cell variance of 10 randomly selected cells of (**a**) ITO/IZO/TaN device and (**b**) ITO/IZO/SiO_2_/TaN device. (**c**) Calculated cell-to-cell variability of devices with and without an SiO_2_ layer.

Figure 2 shows the cell-to-cell variations in both devices. A total of 10 randomly selected cells from both devices were selected, and 20 cycles were run for each cell. The addition of a SiO_2_ layer enhances cell-to-cell and cycle-to-cycle uniformity as illustrated in Figure 2a,b. Figure 2c also depicts the cell-to-cell variability of the ITO/IZO/TaN and ITO/IZO/SiO_2_/TaN devices. By introducing a SiO_2_ thin film, the variability of both HRS and LRS was improved.

**Figure 3:**
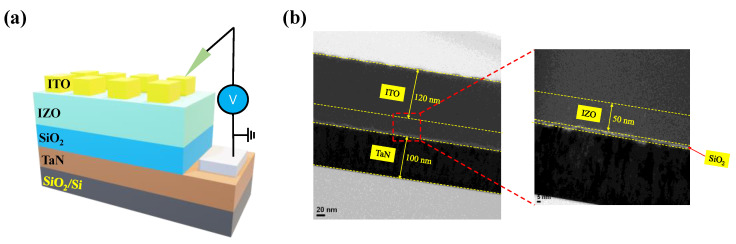
(**a**) Schematic structure of ITO/IZO/SiO_2_/TaN device. (**b**) Cross-sectional TEM images.

**Figure 4:**
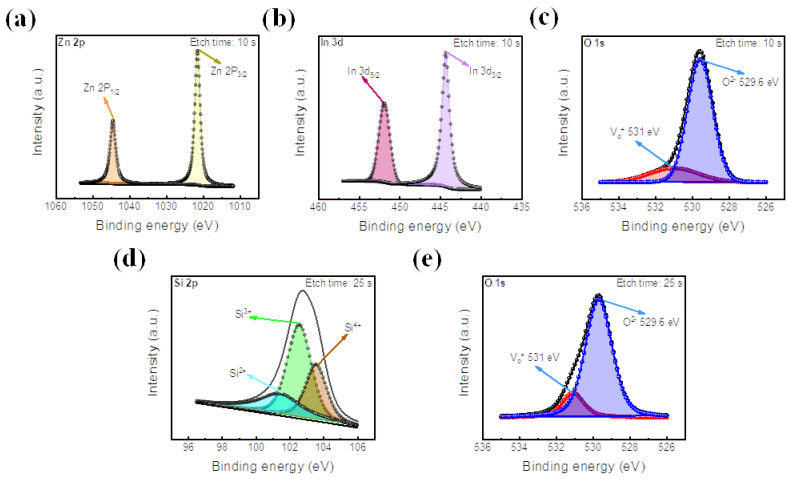
XPS peak analysis of IZO and SiO_2_ films. (**a**) Zn 2p peak, (**b**) In 3d peak, and (**c**) O 1s peak of IZO film. (**d**) Si 2p peak and (**e**) O 1s peak of SiO_2_ film.

To confirm the fabrication of the bilayer device as shown in the schematic structure in Figure 3a, TEM and XPS were used. A cross-sectional TEM image of the device is shown in Figure 3b. The sputter-deposited ITO and TaN electrodes had thicknesses of 120 nm and 100 nm, respectively. Additionally, insulating layers were sandwiched between the electrodes. The thicknesses of the IZO and SiO_2_ layers were about 50 nm and 2 nm, respectively. Furthermore, the XPS depth mode is used to investigate the chemical components of the IZO and SiO_2_ layers, as shown in Figure 4.

XPS spectra of the IZO film are depicted in Figure 4a–c, with an etch time of 10 s. The peak intensity values of Zn 2p are located at the binding energies of 1021.5 eV and 1045.2 eV for Zn 2p_3/2_ and 2p_1/2_, respectively [40]. 3d_5/2_ and 3d_3/2_ are located at about 444.6 eV and 453.35 eV, respectively [67]. In addition, as shown in Figure 4c, the peak of O 1s at 529.6 eV represents metal–oxygen bonding, proving the existence of the IZO insulating layer [68]. Next, the Si-O bond is illustrated at an etch time of 25 s, as shown in Figure 4d,e. The XPS of Si 2p is shown to have three peaks, representing its three oxide states of Si^2+^, Si^3+^, and Si^4+^ [69]. Also, the O 1s peak of Figure 4e is located at about 529.6 eV, demonstrating Si-O bonding. Additionally, in both the O 1s peaks of the IZO and SiO_2_ thin films, an additional peak is located at a binding energy of 531 eV, which represents the existence of oxygen vacancies in each insulating layer [34]. 

**Figure 5:**
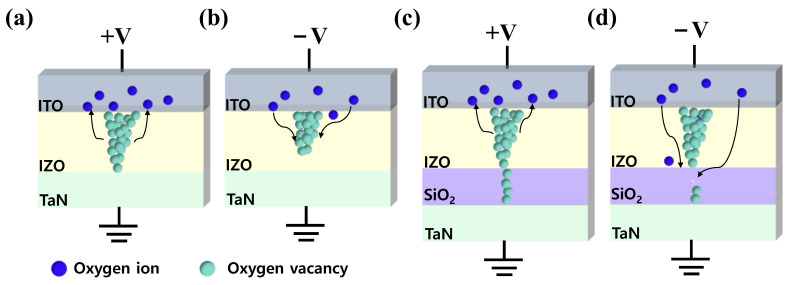
Conduction mechanism of the ITO/IZO/TaN device, (**a**) Set, and (**b**) Reset. Conduction mechanism of the ITO/IZO/SiO_2_/TaN device, (**c**) Set, and (**d**) Reset.

Illustrations of resistive switching mechanisms are shown in Figure 5. For an ITO/IZO/TaN device, when a positive bias is applied to the top electrode, oxygen ions in the IZO film migrate toward the ITO layer via the electric field, where the ITO layer acts as an oxygen reservoir. Then, the amount of oxygen vacancies in the IZO film increases and forms a thick conducting filament, enabling a large current flow in the device. As a result, a set occurs, and the device switches to LRS (Figure 5a). Moreover, when the opposite bias is applied to the top electrode, oxygen ions stored in the ITO layer return to the IZO film and recombine with oxygen vacancies. Thus, a reset occurs due to the rupture of a conducting filament, and the device switches into HRS (Figure 5b). However, due to the random nature of the conducting filament, the single-layer device suffered from poor uniformity. On the other hand, for the ITO/IZO/SiO_2_/TaN device, when a positive bias was applied to the top electrode as shown in Figure 5c, oxygen vacancies generated in the IZO-film accumulated and formed a conical-shaped filament toward the interface of IZO/SiO_2_ [70,71], making the IZO film more conductive [72]. During the formation of the filament, a major drop in electric potential occurred in the SiO_2_ film. A high electric field was applied to the film, localizing the conducting filament and reducing randomness in the formation of the conducting path [44]. Additionally, the reduced current levels were likely achieved due to the narrow thickness of the conducting path in the SiO_2_ film, limiting the current flow. Furthermore, it is believed that when a negative voltage is applied to the top electrode, as illustrated in Figure 5d, the rupture of the conducting filament occurs in the SiO_2_ layer, where, due to its diffusion limiting role, an improvement in uniformity occurs [73,74].

**Figure 6:**
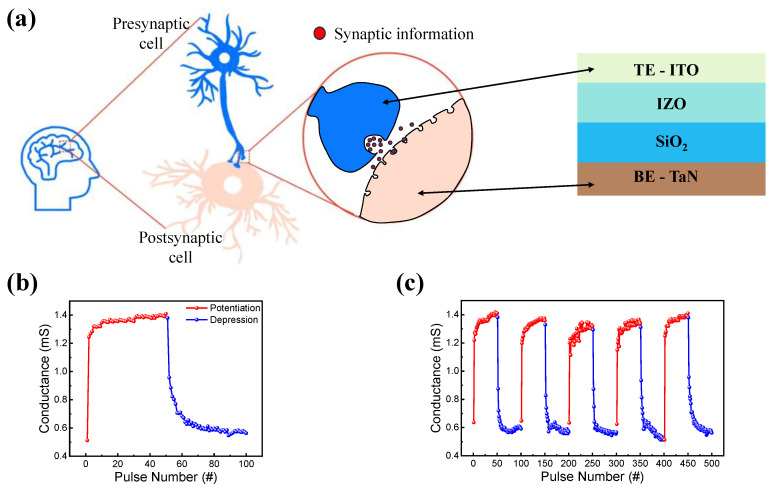
(**a**) Schematic of an RRAM device as a synaptic device and a biological synapse. (**b**) Potentiation and depression. (**c**) Five cycles of repetition of potentiation and depression.

In a biological synapse, synaptic information is processed through neurons. As shown in Figure 6a, neurons are composed of pre- and post-synaptic cells, which can be easily mimicked by a two-terminal RRAM device. As a synaptic device, the top and bottom electrodes mimic pre- and post-synaptic cells. One of the important features of neuromorphic application is potentiation and depression [75]. A pulse train of 50 identical set and reset pulses were used for potentiation and depression. The amplitude and width of the set pulses were 2 V and 0.5 μs, and those of the reset pulse were −1.5 V and 5 μs. Figure 6b shows the increase and decrease in conductance caused by the pulse trains. Five potentiation and depression cycles were also performed to ensure reproducibility, as shown in Figure 6c.

**Figure 7:**
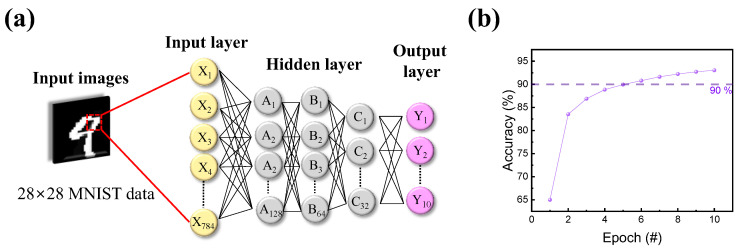
Pattern recognition through a neural network. (**a**) Schematic illustration of a deep neural network for numerical number recognition consisting of input, hidden, and output layers. (**b**) Simulated recognition accuracy using the MNIST numerical data set, with a maximum accuracy of 93.03% for the ITO/IZO/SiO_2_/ITO device.

The results of potentiation and depression were then transformed into an artificial neural network to calculate the accuracy of a Modified National Institute of Standards and Technology (MNIST) handwritten data set. As illustrated in Figure 7a, the deep neural network-based pattern recognition system (PRS) consisted of input, hidden, and output layers. When the 28 × 28-pixel MNIST handwritten data set entered, nodes of layers changed their parameters via the backpropagation method. Then, by repeating its training through epochs, the accuracy of PRS improved with the training time. Figure 7b shows the training outcome after 10 successive epochs, with a maximum accuracy of 93.03% [76].

**Figure 8:**
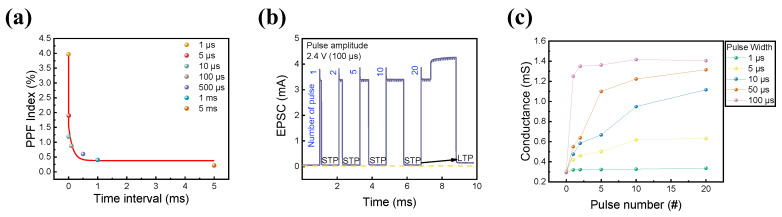
(**a**) PPF as a function of time interval. (**b**) Change in EPSC depending on the number of pulses applied. (**c**) Pulse-width-dependent conductance change.

Furthermore, in the human brain, short-term memory (STM) and long-term memory (LTM) coexist. This was implemented in the short-term device by rehearsing certain events, and STM could be converted into LTM [77]. Thus, the coexistence of STM and LTM characteristics in RRAM devices is beneficial for synaptic applications. PPF is neural facilitation in biological systems related to short-term synaptic plasticity (STP) [78,79,80]. The STP behavior was replicated in the ITO/IZO/SiO_2_/TaN device to investigate the coexistence of volatility and non-volatility in one RRAM cell. A twin pulse with an amplitude of 2.6 V and a width of 10 μs was applied to the semiconductor device under a varied pulse interval of 1 μs to 500 μs. The PPF behavior is known to be related to the negative correlation between time interval and PPF index (PPF index = ((I_2_ − I_1_)/I_1_) × 100, where I_2_ is the current response of the second pulse and I_1_ is the current response of the first pulse train). When the time between two pulses was short, the device remembered the first stimulus, resulting in a greater current response to the second stimulation. When the gap was long enough, however, the system forgot about the first stimulus and no variation occurred in the second pulse. The result of the twin pulse scheme is illustrated in Figure 8a. 

Additionally, the EPSC change in the device by varying pulse number was conducted to check whether the ITO/IZO/SiO_2_/TaN device could convert STM to LTM by repeating the experiment. The pulse amplitude and width were fixed at 2.4 V and 100 μs. The number of pulses varied from 1 to 20. Figure 8b shows that as the number of pulses increased, the EPSC increased, and the read current abruptly surged after 20 consecutive pulses, indicating that the STM was switched to LTM. To further investigate the relationship between pulse width and its current response, the EPSC test was repeated with the pulse width varying from 1 μs to 100 μs, while the pulse amplitude had a fixed value of 2.6 V. The result is shown in Figure 8c, where the linear relationship between pulse and conductance is observed. When the pulse width was short, the STM characteristics remained. On the other hand, when the pulse width was long, long-term potentiation occurred from the first single pulse, resulting in higher conductance at the final 20 consecutive pulses.

**Figure 9:**
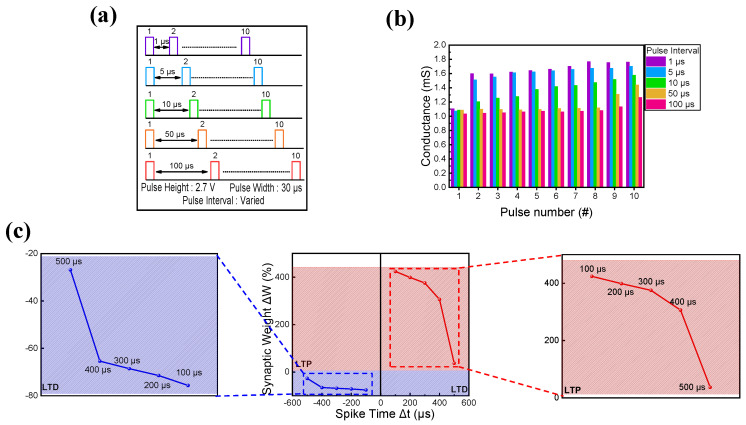
(**a**) Schematic illustration of pulses applied to observe SRDP behavior. (**b**) SRDP and (**c**) STDP behaviors of ITO/IZO/SiO_2_/TaN device.

Finally, Hebbian rules were applied to ITO/IZO/SiO_2_/TaN devices [81]. As the basis of neuromorphic computing implementation is emulating the biological brain, synaptic devices need to mimic the brain’s synapse and neuron properties. In this synapse and neuron interconnection process, various functions occurred under given stimuli, strengthening or weakening the synaptic connection [82]. One of the ways to implement this weight change is by following the Hebbian rules, which are learning tools that verify the ability of the synapse device to imitate the synaptic plasticity of biological synapses. Among various Hebbian rules, SRDP and STDP are two of the most typical ways to copy synaptic weight change and information processing between pre- and post-synaptic cells [83,84,85,86,87]. First, SRDP behavior is observed by altering the pulse interval. The schematic illustration of the pulse train is shown in Figure 9a, where the pulse interval varies from 1 μs to 100 μs. The pulse height and width have fixed values of 2.7 V and 30 μs. In Figure 9b, the SRDP response is illustrated. The pulse interval and conductance value are shown to have a linear relationship. When the pulse interval was short, the synapse received practically consecutive pulse trains, rapidly increasing the conductance. When the pulse interval was long, however, the synapse ‘forgot the previous stimulus, resulting in a gradual increase in conductance. Furthermore, STDP was examined by applying the same pair of pulses to the pre-and post-synapse under varied time difference conditions. Spike time (Δt) was defined as the time difference of pulse trains applied to the pre-and post-synapses (Δt = t_post_ − t_pre_. t_post_ and t_pre_ are the times when pulses are applied to the pre-and post-synapses). If the pre-synapse fired before the post-synapse (Δt > 0), a set of pulses led to LTP. Conversely, if the post-synapse fired before the pre-synapse (Δt < 0), a different set of pulses caused LTD. The STDP behavior of the ITO/IZO/SiO_2_/TaN device is illustrated in Figure 9c, where the depression of the synaptic weight (ΔW) is shown with an increase in spike time value. Here, ΔW = (G_f_ − G_i_)/G_i_) × 100, where G_f_ is the conductance value of the device after pulse application and G_i_ is the conductance value of the device before pulse application.

## 4. Conclusions

In summary, the resistive switching and synaptic properties of IZO-based RRAM devices were investigated by electrical measurements, including DC sweep and pulse response. The bilayer-structured ITO/IZO/SiO_2_/TaN device demonstrated superior memory characteristics compared to the single-layer device, consuming less power and showing better uniformity. It is noted that the inserted SiO_2_ layer prevented the hard breakdown of the IZO layer and localized the conducting filament. Additionally, synaptic functions were assessed using pulse measurements and other biological synapse learning criteria. The PPF, potentiation, depression, and EPSC changes demonstrate the coexistence of STM and LTM. Finally, STDP and SRDP prove the potential of the ITO/IZO/SiO_2_/TaN device to be used in future neuromorphic system applications.

## Data Availability

Data are contained within the article.
